# Rapid Calculation of Residual Stresses in Dissimilar S355–AA6082 Butt Welds

**DOI:** 10.3390/ma14216644

**Published:** 2021-11-04

**Authors:** Francesco Leoni, Hallvard Gustav Fjær, Paolo Ferro, Filippo Berto

**Affiliations:** 1 Department of Mechanical and Industrial Engineering, Norwegian University of Science and Technology, Richard Birkelands vei 2b, 7491 Trondheim, Norway; filippo.berto@ntnu.no; 2 Department of Computational Materials Processing, Institute for Energy Technology, 2027 Kjeller, Norway; Hallvard.Fjar@ife.no; 3 Department of Management and Engineering, University of Padova, Stradella S. Nicola, 2, I-36100 Vicenza, Italy; paolo.ferro@unipd.it

**Keywords:** dissimilar welds, S355, AA6082, residual stresses, rapid calculation, analytical method

## Abstract

An analytical model is proposed to rapidly capture the thermal and residual stresses values induced by the hybrid metal extrusion and bonding (HYB) process on dissimilar-metal butt-welded joints. The power input for two welding velocities is first assessed using a thermal–mechanical model solved by a heat generation routine written in MATLAB code. Subsequently, the obtained temperature history is used as input to solve the equilibrium and compatibility equations formulated to calculate the thermal and residual stresses. To verify the soundness of the analytical approach, a Finite Element numerical model of the entire process is carried out and results are compared with those coming from the proposed rapid method. It is found that the degree of accuracy reached by the analytical model is excellent, especially considering the tremendous time reduction when compared to that characterizing the standard numerical approach.

## 1. Introduction

Metal structures subjected to welding processes are characterized by residual stresses that are a consequence of heating and cooling cycles occurring in the joint and in the parent material. The localized heating induced by welding results in non-uniform temperature fields, which are associated with material thermal expansions and contractions. In the case of butt-welded plates, during the heating phase, compression stresses arise near the joint along the weld line. This happens because the thermal expansion of the hot area is constrained by the mass of the parent material that remains cold. Localized high plastic deformations in the metal, softened by the high temperature, are therefore promoted. If the metal deforms plastically in a non-uniform manner, the elastic recovery during the subsequent cooling phase does not fully occur, and the material remains in a state of residual tension. It is known that residual tensile stresses can contribute to brittle fractures or cause premature failures in components subjected to fatigue, while compression stresses are responsible for deformations and buckling in thin large panels, such as those used in the automotive industry. For these reasons, an assessment of the magnitude and distribution of residual stresses can be extremely helpful in the design phase of a welded structure [1,2].

In the past, welding residual stress investigation employed only experimental measurement methods. These methods can be divided in two categories: destructive and non-destructive. The first category comprises, among the others, hole drilling and strain gauge techniques [3,4,5,6], while non-destructive methods are X-ray diffraction (XRD), neutron diffraction, and ultrasonic waves [6,7,8]. However, all these experimental techniques are time-consuming and unable to capture the complete stress distributions over the entire component geometry. For instance, although the XRD method is highly accurate, it provides limited information about only a relatively thin surface layer [9,10].

In recent years, the development of numerical Finite Element codes made it possible to simulate the whole welding process, considering all the related effects such as the precipitates’ dissolution, solid-state phase transformation, and elastic-plastic and creep behavior of the material under investigation. For example, in his work, Deng [11] used a coupled thermal, metallurgical, and mechanical 3D finite element model to investigate the effects of solid-state phase transformation on welding residual stress and distortion in low carbon and medium carbon steels, revealing that the final residual stress and distortion in low carbon steel did not seem influenced by the solid-state phase transformation, while for the medium carbon steel, the final residual stresses and distortions appeared to be affected by the martensitic transformation. In addition, Chen et al. [12] developed a three-dimensional finite element model to investigate the thermal history and thermomechanical process of friction stir (FS) butt-welded AA6061-T6 plates. They investigated the evolution of longitudinal, transversal, and through-thickness stress and used X-ray diffraction to validate their results. D’Ostuni et al. [13] developed a FE model to simulate dissimilar aluminum–titanium fiber-lased welds employing two-dimensional and three-dimensional Gaussian heat sources. Finally, Obeid et al. [14] developed a 3D FE model to simulate a circumferential single-pass weld overlay and two-pass girth welding and validated the results coming from the model with experimental measurements. In general, numerical methods provide useful and accurate information, but they need a great computational effort and can be relatively time-consuming, depending on the dimension of the joint and the number of welding runs to be simulated.

In the case of thin plates butt-welded, some simplifications are possible, and this allows one to face the problem via analytical approaches. The system of equations governing the phenomenon can be solved by an iterative procedure easily implemented on current computers with programs, allowing drastic reductions in computational effort compared with more sophisticated non-linear FE analyses. The use of analytical models for the study of residual stresses has already been proposed by different authors in the past. Goff [15] simplified the problem by assuming constant thermal properties of the material and a linear temperature distribution in a direction perpendicular to the weld line. Tall [16] used the classical Rosenthal’s solution [17] for the temperature assessment and suggested an iterative method in which for each temperature increase, the thermal stresses needed to be in equilibrium; the results obtained for each temperature increment were then added to the stresses calculated in the previous time interval. Agapakis and Masubuchi [18] extended the Tall’s previous work by solving, with an iterative procedure, the constitutive and equilibrium equations that govern the problem. J. Cañas et al. [19,20] proposed a model in which the plates were divided into bars, and the governing equations were written in matrix form, allowing them to obtain a more efficient calculation algorithm. In all of these works, the welded plates did not present any constraint. Finally, Ferro et al. [21] proposed a more general model for the calculation of residual stresses induced by fusion welding, starting from the model proposed by Cañas et al. [19,20] and extending the model also to the case of fully constrained plates.

Recently, a new solid-state joining method known as the hybrid metal extrusion and bonding (HYB) process has appeared on the horizon [22]. So far, various experimental studies of the HYB process applied to similar and dissimilar metals welds were carried out in order to better understand and predict the effect of process parameters on welded materials; however, only recently was a great effort spent in the development of numerical models [23,24]. Recently, good experimental results were found for dissimilar aluminum–steel butt welds obtained by the HYB technique [25,26,27]. Numerical models were widely used in the literature to study the thermal field and residual stress arising during similar and dissimilar metal welds production; however, analytical investigations on dissimilar welded joints produced through this novel welding technique are still missing in the literature.

The present work is aimed at filling this gap by extending the analytical model proposed by Ferro [21] to dissimilar aluminum–steel butt-welded joints produced via HYB. The equations that solve the problem were written in matrix form and solved by an iterative procedure using MATLAB^®^ code. A 3D finite element model was also developed that uses a double ellipsoid volume function with Gaussian power distribution to simulate the moving heat source. Results coming from the two different approaches, numerical and analytical, were finally compared with each other with the goal of verifying the soundness of the analytical method.

## 2. Materials and Methods

### 2.1. Geometry and Materials

**Figure 1:**
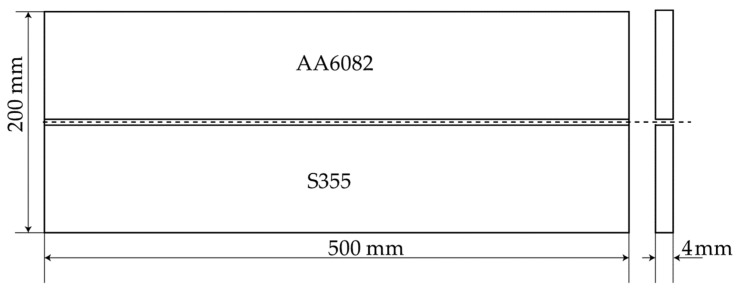
Geometry of the welded plates.

A 4 mm thick aluminum alloy AA6082 plate butt-welded to a 4 mm thick structural steel S355 plate was considered. All dimensions are shown in Figure 1.

**Figure 2:**
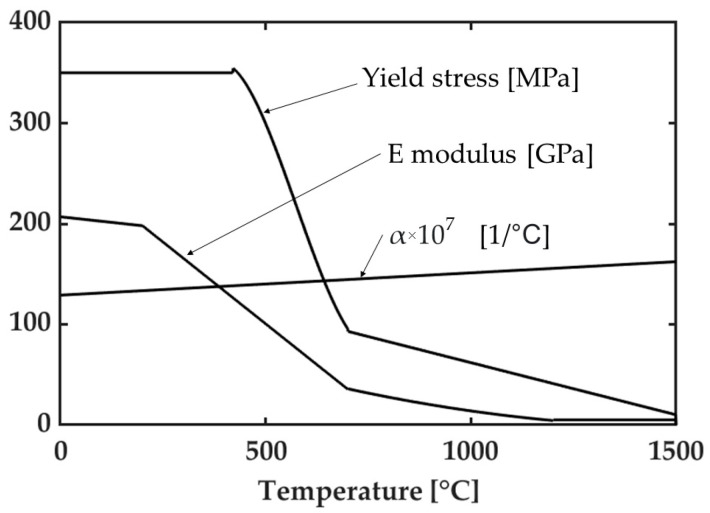
Material properties of S355 steel [28].

**Figure 3:**
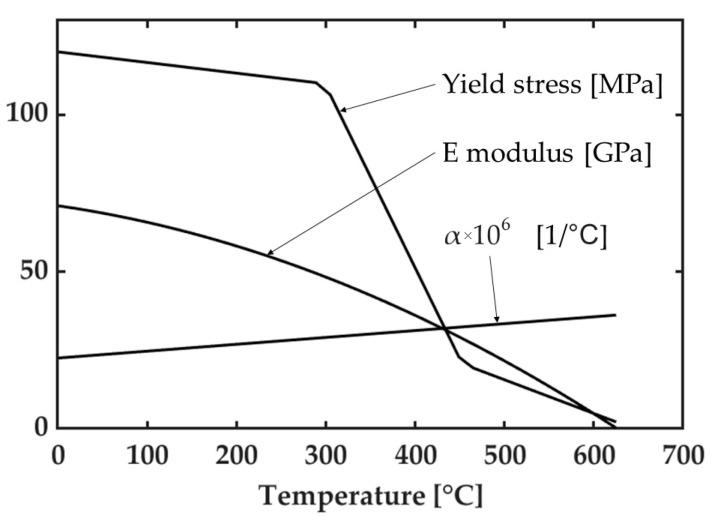
Material properties of AA6082 aluminum alloy.

Materials properties, as a function of temperature, are shown in Figure 2 and Figure 3. More in detail, thermal properties were kept constant with temperature since previous studies have shown that this simplifying hypothesis gives acceptable results when considering the residual stress computation. The thermal conductivity, thermal diffusivity, and melting temperature of the aluminum alloy were set equal to 170 W/(mK), 62 mm^2^/s, and 638 °C, respectively. On the other side, with reference to same thermal properties, their values for the steel were 44 W/(mK), 11.5 mm^2^/s, and 1510 °C. The Poisson’s ratio was also kept constant with temperature and equal to 0.34 and 0.28 for aluminum and steel, respectively.

Material properties as a function of temperature can be also written in closed form as in Appendix A.

For the sake of simplicity, microstructural changes, with particular reference to secondary phases evolution in AA6082 aluminum alloy, were not considered herein. However, their effects on thermal and residual stresses were implicitly taken into account by temperature-dependent mechanical material properties.

### 2.2. The HYB Process

**Figure 4:**
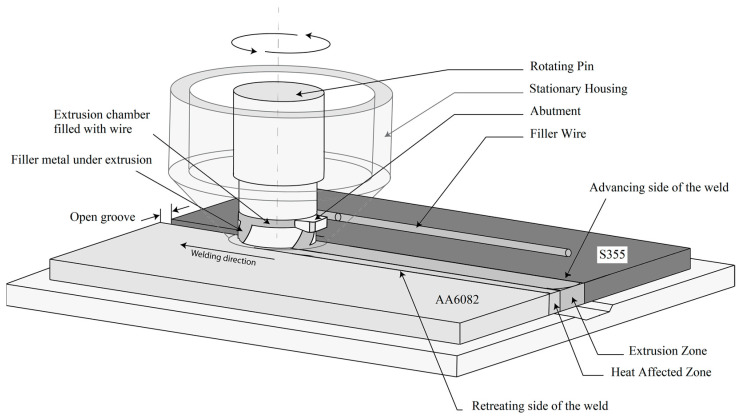
Schematic illustration of the HYB process applied to dissimilar metals with its main parts being involved in the joining process.

The HYB process is based on the principles of continuous extrusion and allows joining to be performed using aluminum filler metal (FM), similar to gas metal arc welding (GMAW) but without any melting involved. Figure 4 highlights the core parts forming the HYB PinPoint extruder. In the situation of butt-welding, the two plates to be joined are divided from each other by a fixed spacing so that an I-groove forms between them. During the welding operation, the PinPoint extruder shown in Figure 4 moves along the joint line at a constant travel speed, while the rotating pin with its moving dies is submerged between the plates. This enables the extrudate to flow downwards in the axial direction and mix with the base metal (BM) in the groove under conditions of high pressure and severe plastic deformation. Metallic bonding between the FM and the BM occurs as a result of these two contributions, along with oxide dispersion [22]. By taking into consideration the mass balance involved, the extrudate flow rate can be adjusted (by using the rotational speed of the drive spindle as the main process variable) in such a way that the groove between the plates is filled with solid aluminum in a continuous manner. More information about the HYB welding technique can be found elsewhere [25,29,30].

## 3. Modelling

**Figure 5:**
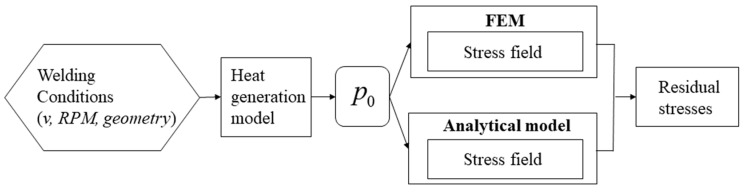
Flowchart showing the steps involved in calculating the net power input using the heat generation model and the subsequent Finite Element and analytical/numerical analysis for each corresponding welding condition.

Figure 5 shows a flowchart of the numerical/analytical work proposed. As a starting point, the welding conditions such as welding speed, rotational speed, and geometry of the tool were fixed. These input parameters were used to calculate the corresponding heat input by using a semi-analytical heat generation model already proposed by Leoni et al. [24]. The obtained heat input was then used both in FE and analytical model to calculate the thermal and residual stresses.

Table 1 shows the welding conditions investigated.

### 3.1. Thermal FE Model

**Figure 6:**
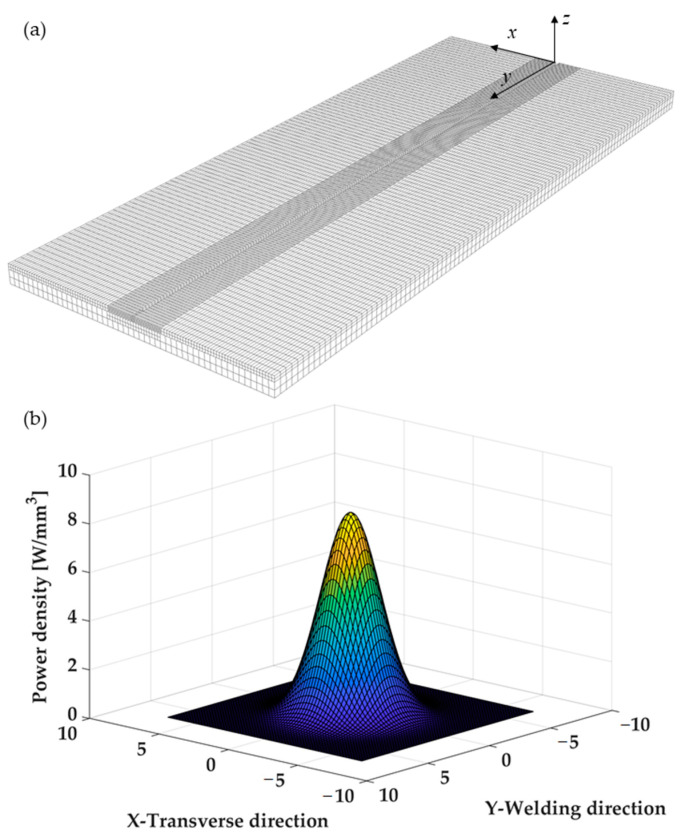
(**a**) Mesh used for the FE simulations. (**b**) Heat flux at top layer (z = 0) and in the local reference system using the double ellipsoid power density distribution function, as proposed by Goldak and using the following parameters: $p_{0}$ = 1000 W, $k_{1}$= 4 mm, $k_{2}$= 4 mm, $k_{3,f}=k_{3,r}=$ 5 mm.

The numerical model was developed using WELDSIM Finite Element code. The geometry was modeled with 47,032 and 17,000 hexahedral finite elements for the plates and the backing plate, respectively. Moreover, a finer mesh was created in the vicinity of the weld line in order to capture the high temperature gradients that characterize that region (Figure 6a) with the smallest element size of 0.75 mm. The effect of the filler material was also taken into account by element activation; finally, the moving heat source was modeled as a double ellipsoid power density distribution function, as proposed by Goldak [31]. The power density of the double-ellipsoid heat source describing the heat flux distribution in the front and rear of the heat source can be expressed as follows:

(1) $$p_{f}(x,y,z,t)=\frac{6\sqrt{3}p_{0}}{k_{1}k_{2}k_{3,f}\pi \sqrt{\pi }}e^{-3x^{2}/k_{1}{}^{2}}e^{-3z^{2}/k_{2}{}^{2}}e^{-3{\left(y+v(\tau -t)\right)}^{2}/k_{3,f}{}^{2}}$$

(2) $$p_{r}(x,y,z,t)=\frac{6\sqrt{3}p_{0}}{k_{1}k_{2}k_{3,r}\pi \sqrt{\pi }}e^{-3x^{2}/k_{1}{}^{2}}e^{-3z^{2}/k_{2}{}^{2}}e^{-3{\left(y+v(\tau -t)\right)}^{2}/k_{3,r}{}^{2}}$$

where *x*, *y*, and *z* (mm) are the coordinates as in Figure 6a, $k_{1}$, $k_{2}$, $k_{3,f}$, $k_{3,r}$ (mm) are the half-width, the depth, and the front and rear length of the double ellipsoid heat source, respectively, τ (s) is the lag time, $p_{0}$ (W) is the net power input, and v (mm/s) the welding speed.

The heat transfer coefficient with the backing plate was considered to be 200 W/m^2^ °C, while convective heat loss was modeled using a convective heat transfer coefficient equal to 20 W/m^2^ °C, in agreement with a high air-flow assumption [32].

### 3.2. Thermal Analytical Model

The Rosenthal’s equation was used for the rapid thermal field calculation in the analytical model:

(3) $$T=T_{0}+\frac{p_{0}/d}{2\pi \lambda }\exp\left[-\frac{vy}{2\alpha }\right]K_{0}\left(\frac{vr}{2\alpha }\right)$$

In Equation (3), $T_{0}$ is the reference temperature set equal to 20 °C, y is the coordinate of a point of the plate in the same direction of the weld line, r is the distance from the heat source center, v is the welding speed, $K_{0}$ is the modified Bessel function of the second type and zero order, λ is the thermal conductivity, and α is the diffusivity. The Rosenthal’s Equation (3) is based on the following assumptions [33]:

- the plates are considered semi-infinite and of thin thickness;
- the equation describes the temperature range induced by a linear source: temperature gradient along the thickness of the plates is neglected;
- the thermal field refers to a quasi-stationary condition of the welding process.

It is worth noting that $p_{0}$, the actual thermal power of the pin, is calculated employing the model described by Leoni et al. [24] and summarized with the following equation:

(4) $$p_{0}=p_{0}\left(\omega \;,\tau_{m},T_{\mathrm{int}},v,r_{tip},r_{sh},d,h_{1},h_{2},\overset{\cdot }{m}\;,T_{FM}\right)$$

where *ω* (rad/s) is the angular velocity of the tool, $\tau_{m}$ (MPa) is the shear stress at the tool-matrix interface, $T_{\mathrm{int}}$ (°C) is the temperature at the tool-matrix interface, v (mm/s) is the welding speed, $r_{tip}$ (mm) is the radius of the tip of the tool, $r_{sh}$ (mm) is the radius of the shoulder of the tool, d (mm) is the thickness of the plates, $h_{1}$ (mm) is the difference between the radius of the tip and half width of the groove, $h_{2}$ (mm) is the difference between the radius of the shoulder and half width of the groove, $\overset{\cdot }{m}$ (kg/s) is the flow of the hot filler material being extruded, and $T_{FM}$ (°C) is the temperature of the hot extrudate. Equation (4) describes the physical framework of the heat generation during the hybrid metal extrusion and bonding process (i.e., the extrusion of filler material and the friction between the tool and the base material). It is noted that the coupling between the mechanical (shear stress) and thermal field (*T*) (Equation (3)) is solved by an iterative routine written in MATLAB code and described elsewhere [24].

## 4. Residual Stress Assessment

### 4.1. Finite Element Analysis

**Figure 7:**
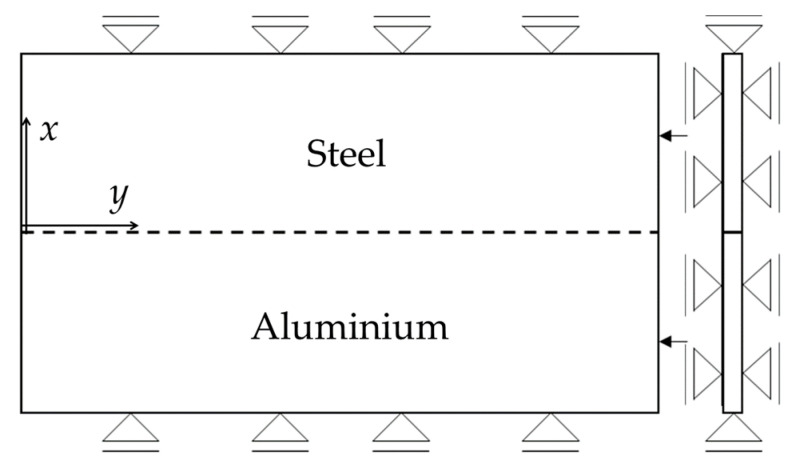
Schematic representation of the welded plates with lateral and out-of-plane constraints (uniformly applied to the top and bottom surfaces of the plates) assumed in the FE model.

Following the computational welding mechanical (CWM) approach, the temperature history at each node of the model was used as load for the mechanical computation. The mechanical boundary conditions are summarized in Figure 7 (*U_x_* = 0 for *x* = 0 and for *x = 2B*, where *U_x_* is the displacement in *x* direction, and *B* is the plate width). The plates were left free to deform in the *y* direction. In addition to the in-plane boundary constraints, in the FE model, the out of plane movement of the plates was constrained (Figure 7). The interface between the two plates was modelled as perfectly bonded as the weld proceeded. A sequential and uncoupled thermal and mechanical simulation was used since the thermo-mechanical coupling was already considered in the previous power input calculation (Equation (4)). It is worth noting that the materials constitutive laws were kept the same for both the numerical and analytical model. In particular, for the sake of simplicity, an elastic perfectly plastic law was used for the two materials to be welded.

### 4.2. Analytical Model

**Figure 8:**
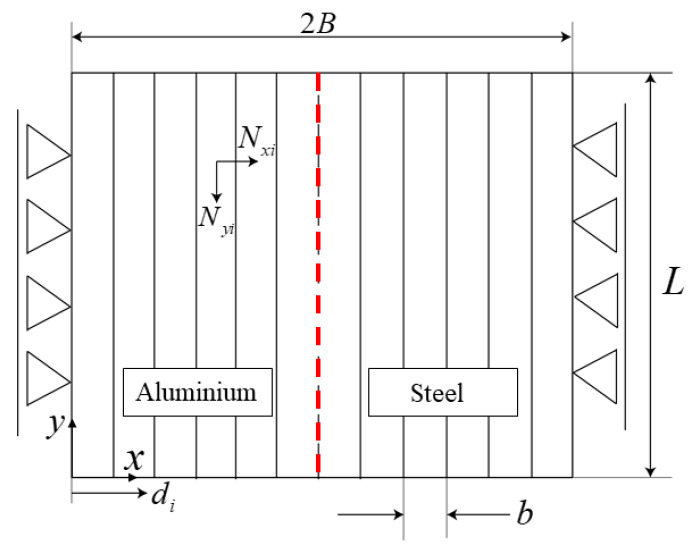
Schematic representation of the welded plates with lateral constraints and weld line highlighted in red.

With reference to Figure 8, in order to write the equations that govern the problem in matrix form (constitutive, equilibrium, and compatibility equations), the welded plates were divided into a discrete number (*n*) of bars. For the sake of simplicity, all bars had the same width (*b*), while *n* was chosen sufficiently high to account for the temperature gradient near the weld line with a good approximation. It was assumed that at each time step the temperature of each bar was uniform, depending only on its *x* coordinate (i.e., *T = T (x, t*)). In other words, the temperature gradient in the *y* direction was neglected, a hypothesis that tends to be valid more and more as the welding speed increases. As for the FE model, boundary conditions were applied at the plate ends according to the HYB process (*U_x_* = 0 for *x* = 0 and for *x = 2B*, where *U_x_* is the displacement in *x* direction, and *B* is the plate width, while the boundary conditions in *y* direction are described by the compatibility Equation (11)). The Von Mises yield criterion was used, and materials viscous-plastic effects were neglected; the yield stress, the elastic modulus, and the thermal expansion coefficient of both materials were considered functions of temperature, as described by Equations (A1)–(A14).

If $N_{y,i}$ and $N_{x,i}$ are the forces on cross sections in bar *i*-th originating from the internal stresses and normal to the *x* and *y* axes, respectively, the equilibrium equations of forces and momentum can be written in matrix form (Figure 8) as follows [21]:

(5) $${\bar{C}}^{T}{\bar{N}}_{y}=\bar{0}$$

(6) $$N_{xi}=N_{x}\;for\;i=1\ldots n$$

where

(7) $$\bar{C}=\left[\begin{array}{cc} 1 & d_{1}\\ \ldots & \ldots \\ 1 & d_{i}\\ \ldots & \ldots \\ 1 & d_{n} \end{array}\right]$$

(8) $${\bar{N}}_{y}=\left[\begin{array}{c} N_{y1}\\ \ldots \\ N_{yi}\\ \ldots \\ N_{yn} \end{array}\right]$$

where *n* is the number of discrete bars, and $d_{i}$ is the *x*-distance of bar *i*-th from the origin (Figure 8).

Under the hypothesis of materials elastic perfectly plastic behavior, the constitutive equations are:

(9) $${\bar{q}}^{y}={\bar{q}}_{e}^{y}+{\bar{q}}_{t}^{y}+{\bar{q}}_{p}^{y}+{\bar{\Delta q}}_{p}^{y}$$

(10) $$q_{i}^{x}=q_{ei}^{x}+q_{ti}^{x}+q_{pi}^{x}+\Delta q_{pi}^{x}\;for\;i=1\ldots n$$

where ${\bar{q}}^{y}$ is the vector containing the total elongations of each discrete bar for the longitudinal direction; $q_{ei}^{y}$ is the elastic contribution to the elongation of the *i*-th bar governed by Hooke’s law; $q_{ti}^{y}$ is the elongation of the *i*-th bar due to the thermal expansion; $q_{pi}^{y}$ is the plastic deformation accumulated in the previous time steps for the *i*-th bar; $\Delta q_{pi}^{y}$ is the plastic deformation of the *i*-th bar at current time step; $q_{i}^{x}$ is the total elongation of the *i*-th bar in the *x* direction, $q_{ei}^{x}$ is the elastic contribution to the elongation in *x* direction of the *i*-th bar, and it is governed by Hooke’s law; $q_{ti}^{x}$ is the elongation in *x* of the *i*-th bar due to the thermal expansion; $q_{pi}^{x}$ is the plastic deformation in *x* direction for the *i*-th bar accumulated in the previous time steps; $\Delta q_{pi}^{x}$ is the plastic deformation of the *i*-th bar in *x* direction calculated at the current time step.

**Figure 9:**
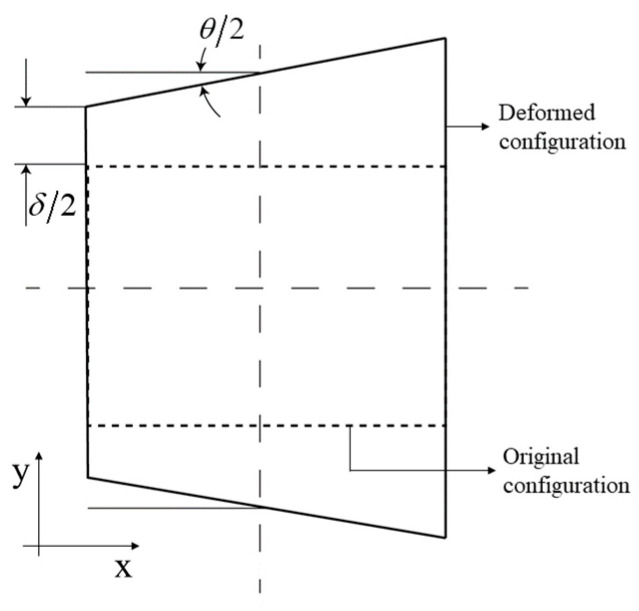
Schematic representation of the deformed and undeformed configuration for the welded plates.

Finally, the compatibility equations are:

(11) $${\bar{q}}^{y}=\bar{C}\bar{u}$$

(12) $$\sum_{i=1}^{n}q_{i}^{x}=0$$

where $\bar{u}$ is the displacement vector associated with the degrees of freedom δ and θ, as shown in Figure 9.

Using the Von Mises criterion, the material yields when:

(13) $$\sigma_{VM}=\sigma_{yield}$$

where $\sigma_{yield}$ is the yield stress of the alloy (i.e., S355 or AA6082) at the corresponding temperature.

Equation (9) can be written as follows:

(14) $${\bar{q}}^{y}=\bar{A}{\bar{N}}_{y}-{\bar{A}}_{x}+{\bar{A}}_{t}(\bar{T}-{\bar{T}}_{0})+{\bar{q}}_{p}^{y}+{\bar{\Delta q}}_{p}^{y}$$

where $\bar{A}$ is a diagonal matrix whose terms are ($L/(E\left(T_{i}\right)db)$) (where $T_{i}$ is the temperature of the *i*-th bar at the time step considered), $\bar{A_{x}}$ is a vector in which each element is represented by the term ($\nu N_{x}/(E\left(T_{i}\right)d\;)\;$), $\bar{A_{t}}$ is another diagonal matrix whose terms are $L\alpha \left(T_{i}\right)$, and $\bar{T}$ is the vector containing the temperatures for each bar.

Using Equations (11) and (14), the vector $\bar{N_{y}}$ can be expressed by the equation:

(15) $${\bar{N}}_{y}={\bar{A}}^{-1}(\bar{C}\bar{u}+{\bar{A}}_{x}-{\bar{A}}_{t}(\bar{T}-{\bar{T}}_{0})-{\bar{q}}_{p}^{y}-{\bar{\Delta q}}_{p}^{y})$$

And from the combination of (15) and (5):

(16) $$\bar{u}={\left[{\bar{C}}^{T}{\bar{A}}^{-1}\bar{C}\right]}^{-1}{\bar{C}}^{T}{\bar{A}}^{-1}(-{\bar{A}}_{x}+{\bar{A}}_{t}(\bar{T}-{\bar{T}}_{0})+{\bar{q}}_{p}^{y}+{\bar{\Delta q}}_{p}^{y})$$

Finally, from Equations (10) and (12), one can obtain the following equation:

(17) $$N_{x}=\frac{1}{\frac{b}{dL}\sum \frac{1}{E(T_{i})}}\left(\frac{v}{b}\sum \frac{N_{i}^{y}}{E(T_{i})}-b\sum \alpha (T_{i})(T_{i}-T_{0})-\sum \left(q_{pi}^{x}+\Delta q_{pi}^{x}\right)\right)$$

The unknown terms were $\Delta q_{pi}^{y}$ and $\Delta q_{pi}^{x}$. The calculation routine started by assuming the increase in plastic deformation ($\Delta q_{pi}^{y}$) as well as the force in the transverse (*x*) direction to be initially null. Using Equation (3), the temperature vector (temperature of each bar) was calculated, and therefore the material properties (which depend on temperature), and matrixes $\bar{A}$, $\bar{A_{x}}$, and $\bar{A_{t}}$. Under such conditions, a first value of $\bar{N_{y}}$, using Equations (15) and (16), was determined. The following condition was then imposed:

(18) $$\left|N_{i}^{y}\right|\leq N_{pi}\;for\;i=1\ldots n$$

where $N_{pi}$ indicates the yield force of the *i*-th bar (temperature dependent). It was then possible to calculate a first value of the plastic elongation ($\Delta q_{pi}^{y}$) by using the previous value of the vector $\bar{u}$ and Equations (11) and (14).

This first approximation of plastic elongation was then used in (15), (16), and (18) for a second approximation of the vector $\bar{N_{y}}$, and such procedure was repeated until convergence was achieved for the time step considered.

Subsequently, a first approximation of *N_x_* was calculated with Equation (17), using the values of $N_{i}^{y}$ from the previous step and assuming to be null the increase of plastic deformation ($\Delta q_{pi}^{x}$).

After having imposed the following condition (19) (derived from (13)),

(19) $${\left[{\left(\frac{N_{x}}{dL}\right)}^{2}+{\left(\frac{N_{i}^{y}}{db}\right)}^{2}-\frac{N_{x}N_{i}^{y}}{d^{2}Lb}\right]}^{0.5}\leq \sigma_{yield,i}$$

and using Equation (17), a first approximation of the term $\Delta q_{pi}^{x}$ was obtained, and this was used for a second approximation of *N_x_*; this was repeated until convergence was achieved. The value of *N_x_* was then used to determine the new value of $\bar{N_{y}}$, and if the last value was not equal to the previous one (within the tolerance imposed), *N_x_* and $\Delta q_{pi}^{x}$ were recalculated using the last obtained value of $\bar{N_{y}}$; this iteration was repeated until convergence was achieved.

Ferro et al. [21] showed that the term coupling the longitudinal with the transversal stress (i.e., ν*N_x_/L*) is much smaller than the main term, $N_{i}^{y}/b$, and that the transversal stress is no longer negligible only below 300 °C, where the yield strength is not significantly affected by the temperature (Figure 3). This means that, at each time step, the first calculated value of *N_x_* does not have a significant influence on the vector $\bar{N_{y}}$ obtained by assuming initially *N_x_* = 0, and therefore $\bar{N_{y}}$ is limited only by the yield stress of the material.

**Figure A1:**
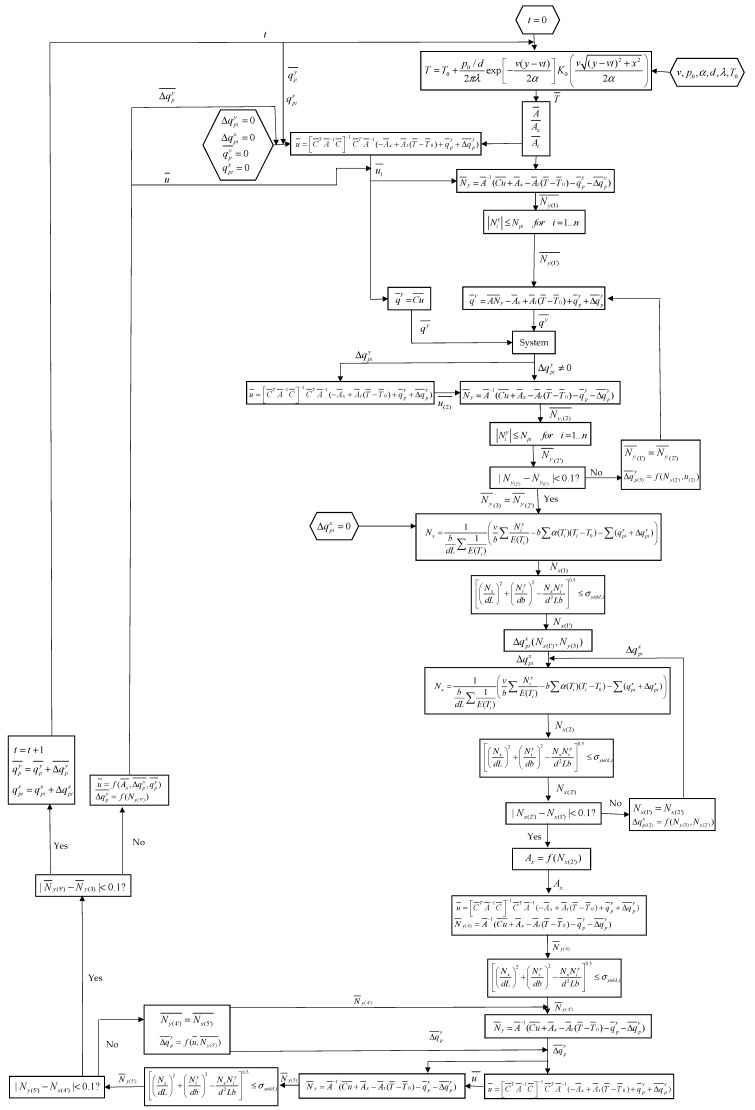
Flow chart of the analytical model.

The algorithm described (see also Figure A1 in Appendix A) was written in MATLAB^®^ code. The input data required by the program were the geometry of the tool (radius of the pin and shoulder), the rotational speed of the tool (ω), the welding speed (v), the plate size (L, 2B, d), the groove width, and the total number of bars (n) (in this work, b = 1 mm, while the time step (Δt) was set equal to 1 s).

## 5. Results

### 5.1. Temperature Results

**Figure 10:**
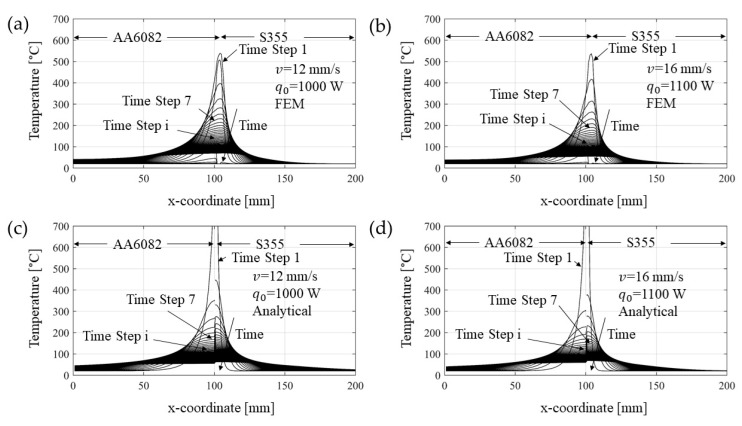
Temperature at different time steps for the welding speed of (**a**,**c**) 12 mm/s and (**b**,**d**) 16 mm/s and power input of (**a**,**c**) 1000 W and (**b**,**d**) 1100 W, respectively, calculated via (**a**,**b**) FE model and (**c**,**d**) via rapid analytical method. Temperatures values were taken considering the mid-thickness of a cross section taken orthogonal to the welding direction in the middle length of the plates.

**Figure 11:**
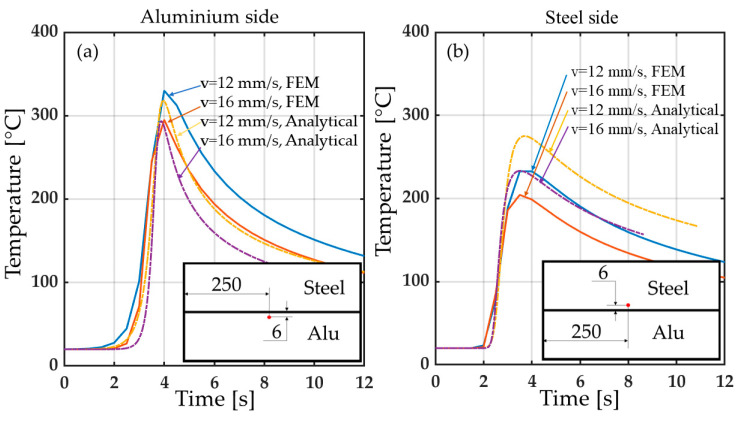
Temperature–time plots exported from two points at the same distance from the weld line (i.e., 6 mm, see schematic representation at the bottom graphs) considering the two welding conditions (i.e., welding speeds of 12 and 16 mm/s and power inputs of 1000 and 1100 W, respectively) for both (**a**) aluminum and (**b**) steel sides.

In Figure 10, the analytical solution of the temperature evolution along the direction perpendicular to the weld line and at different distances from the heat source is compared with that coming from FE model. The results of the two models showed good agreement despite some small differences in the weld line where the Rosenthal’s solution (asymptotic) overestimates the temperature field. However, it is known that no melting occurs during the process [26]; therefore, the latent heat was not considered in the simulations. Moreover, since the analytical solution was considered uncoupled for aluminum and steel, a discontinuity at the interface is observed. In Figure 11 the temperature evolutions at points taken at the same distance from the weld line for both sides are shown. As expected, the analytical solution approximates better the temperature at the aluminum alloy side since it is already known that the Rosenthal’s equation is more suitable for materials that have high thermal conductivity and thermal diffusion coefficients [33]. The differences between analytical and FE results may be due to the natural simplifications required by the Rosenthal’s equation solution, such as the semi-infinite plate geometry deriving from the quasi-stationary thermal field distribution hypothesis. However, despite these apparent discrepancies between the temperatures of the two approaches, the results are in overall agreement, especially if one considers that the most important effect comes from the distribution of the temperature, which finds good agreement between the two methods.

### 5.2. Thermal and Residual Stress Results

**Figure 12:**
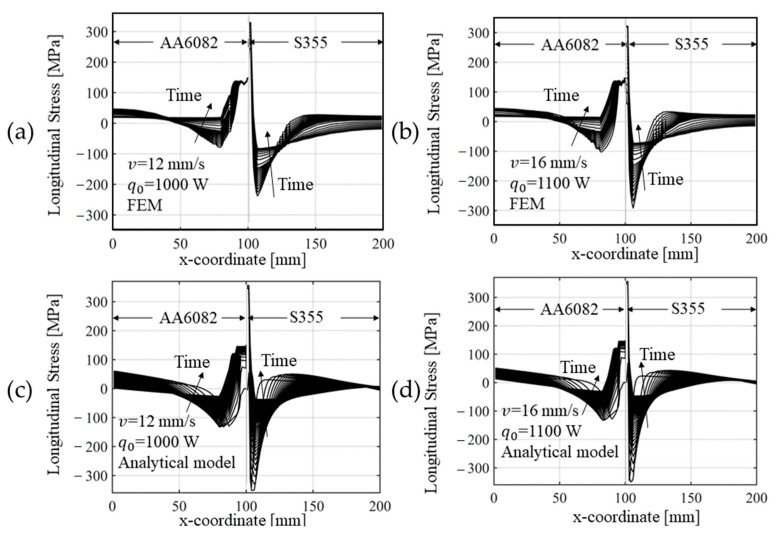
Longitudinal (*y* direction) stress at different time steps for the welding speed of (**a**,**c**) 12 mm/s and (**b**,**d**) 16 mm/s and power input of (**a**,**c**) 1000 W and (**b**,**d**) 1100 W, respectively, calculated via (**a**,**b**) FE model and (**c**,**d**) via rapid analytical method. Values of stress refer to the mid-thickness of a cross section taken orthogonal to the welding direction in the middle length of the plates.

Figure 12 compares the evolution of the longitudinal stress obtained with the numerical and the analytical method proposed. For the comparison with the analytical model, FEM results were taken from the mid-thickness of a cross section at the mid-length of the plates. Because the plates were thin, the residual stress was assumed constant throughout the thickness. A good match was found, and the validity of the procedures adopted, as well as the hypotheses formulated, were confirmed.

**Figure 13:**
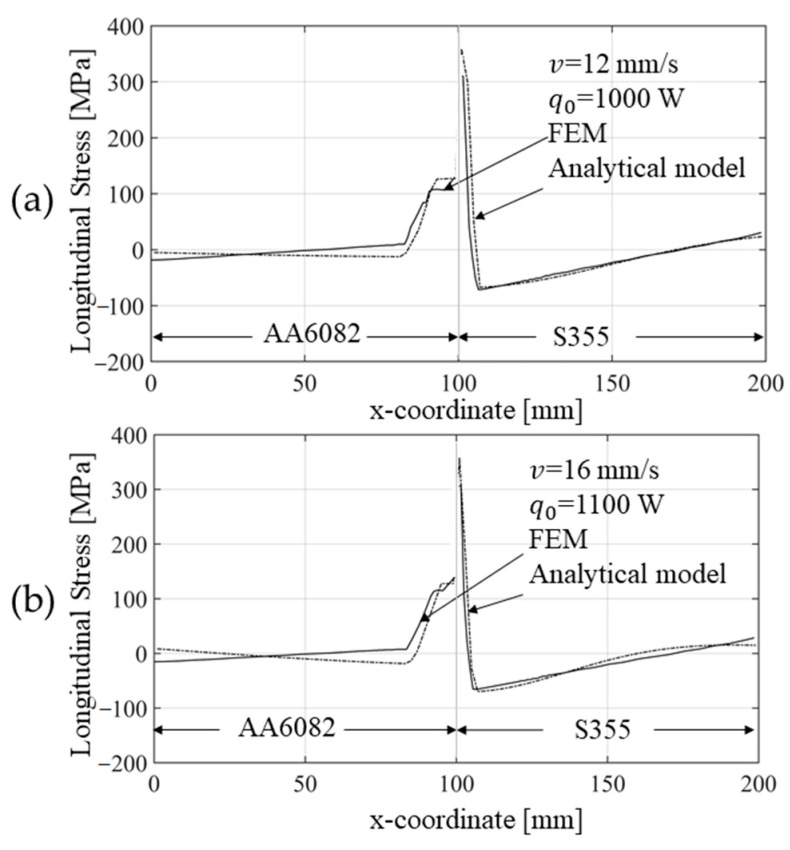
(**a**) Comparison between the residual longitudinal (*y* direction) stress calculated with the two methods (i.e., FEM and analytical model), considering a cross section taken at the mid-length of the plates and for a welding speed of 12 mm/s and a power input of 1000 W. (**b**) Comparison between the residual longitudinal stress calculated with the two methods (i.e., FEM and analytical model), considering a cross section taken at the mid-length of the plates and for a welding speed of 16 mm/s and a power input of 1100 W.

In more detail, the curve showing the longitudinal residual stress value as a function of the transversal direction (*x)* coming from the analytical model overlaps with that obtained using the FE model (Figure 13). At the interface between the two materials, a discontinuity is observed, which is due to the different yield stresses characterizing the two materials. Again, good agreement was observed for both the chosen welding speeds. The slightly wider plasticized zone found with the analytical solution, steel side, can be attributed to the temperature value in that zone, which tends to be overestimated by the Rosenthal’s equation with respect to the more accurate FE calculation (Figure 13). Except for this minor difference, the solutions were in good agreement.

Finally, a comparison between the computational time required by the two methods is presented in Table 2 (RAM: 32 GB, Processor: Intel^®^ Xeon^®^ E-2174G CPU 3.8 GHz)

## 6. Conclusions

A rapid analytical model for the prediction of residual stresses was developed. The hypotheses on which the analytical model formulation is based were validated by comparing the results obtained with a more accurate FE analysis. The numerical analysis was carried out using a 3D model and a double ellipsoid heat source with Gaussian power distribution, while the Rosenthal’s equation was used to calculate the thermal field in the analytical model. In the numerical analysis a thermal source was applied along the welding line, having a constant travel speed, while in the analytical model it was assumed that at each time step each section parallel to the weld line was subjected to the same temperature across its entire length (*T = T (x, t*)). Despite this substantial difference, a good agreement was found between the thermal and mechanical results coming from the two approaches. The simplified model was found to be more advantageous in terms of computational time; it allows one to carry out parametric studies, and it can conceivably be further developed to analyze the effects of different welding setup or stress relief heat treatments on the residual stresses induced by the welding process. In conclusion, although the FE analysis allows one to avoid drastic simplifying hypotheses and provides a series of more detailed information, the proposed simplified method becomes more attractive when a rapid estimation of the thermal and residual stress arising in dissimilar metal welds is required.

## Figures and Tables

**Table 1:** Welding conditions modelled.

Materials	Welding Technique	Welding Speed	Pin Rotational Speed
AA6082–SS355	HYB	12 and 16 mm/s	350 RPM

**Table 2:** Computational time required by the methods used.

FEM	Analytical
64,111 s (~18 h)	~10 s

## Data Availability

Not applicable.

## List of Symbols

**Symbol**	**Value**	**Description**
$\bar{A}$	*E* from Equations (A5)–(A8), Equation (A13), *L* = 500 mm *d* = 4 mm *b* = 1 mm	Diagonal matrix containing ($L/(E\left(T_{i}\right)db)$) terms
$\bar{A_{t}}$	*L* = 500 mm *α* from Equations (A9) and (A14) *T* from Equation (3)	Diagonal matrix containing ($L\alpha \left(T_{i}\right)$) terms
$\bar{A_{x}}$	*υ*_*steel*_ = 0.28 *υ*_*alu*_ = 0.34 *E* from Equations (A5)–(A8) and (A13) *d* = 4 mm	Diagonal matrix containing ($\nu N_{x}/(E\left(T_{i}\right)d)$) terms
B	100 mm	Width of the plate, mm
b	1 mm	Width of the discrete bars used in the analytical model, mm
$\bar{C}$	Equation (7)	Vector containing the values of the distance of each bar from the origin
d	4 mm	Plates thickness, mm
$E_{Alu}$	Equation (A13)	Elastic modulus of aluminum, MPa
$E_{Steel}$	Equations (A5)–(A8)	Elastic modulus of steel, MPa
$h_{1}$	0.75 mm	Difference between the radius of the tip of the tool and half width of the groove, mm
$h_{2}$	3.5 mm	Difference between the radius of the shoulder of the tool and half width of the groove, mm
$K_{0}$	Function	Bessel function of the second kind and zero order
$k_{1}$	4 mm	Half-width of welding pool for the Goldak’s equation, mm
$k_{2}$	4 mm	Depth of welding pool for the Goldak’s equation, mm
$k_{3,f}$	5 mm	Half-length of welding pool for the Goldak’s equation, mm
$k_{3,r}$	5 mm	Half-length of welding pool for the Goldak’s equation, mm
L	500 mm	Length of the plates, mm
$\overset{\cdot }{m}$	5.2 × 10^−4^ kg/s 6.9 × 10^−4^ kg/s	Mass flow of the extrudate, kg/s
n	200	Number of discrete bars
$N_{x}$	Equation (17)	Force in *x* direction, N
$\bar{N_{y}}$	Equation (15)	Vector containing the forces in *y* direction for each bar, N
$p_{0}$	1000 and 1100 W	Power input, W
$p_{f}$	Equation (1)	Power density of the double-ellipsoid heat source, front, W/mm^3^
$p_{r}$	Equation (2)	Power density of the double-ellipsoid heat source, rear, W/mm^3^
$q_{e,i}^{x}$	Calculated	Elastic deformation of *i*-th bar in the *x* direction, mm
$\bar{q_{e}^{y}}$	Calculated	Vector containing the elastic deformations in the *y* direction, mm
$q_{i}^{x}$	Calculated	Deformation of *i*-th bar in the *x* direction, mm
$q_{p,i}^{x}$	Calculated	Plastic deformation of the *i*-th bar in the *x* direction, mm
$\bar{q_{p}^{y}}$	Calculated	Vector containing plastic deformations in the *y* direction, mm
$q_{t,i}^{x}$	Calculated	Thermal deformation of the *i*-th bar in the *x* direction, mm
$\bar{q_{t}^{y}}$	Calculated	Vector containing the thermal deformations in the *y* direction, mm
$\bar{q_{y}}$	Calculated	Vector containing the deformations in the *y* direction, mm
r	Generic point	Distance of a generic point from the heat source center, mm
$r_{sh}$	5.5 mm	Pin shoulder radius, m
$r_{tip}$	2.75 mm	Pin tip radius, m
T	Equation (3)	Temperature, °C
$\bar{T}$	From Equation (3)	Vector containing the temperature of each bar, °C
$T_{0}$	20 °C	Reference temperature, °C
${\bar{T}}_{0}$	Vector of *T*_0_	Vector containing the reference temperature of each bar, °C
$T_{FM}$	450 °C	Temperature of the hot extrudate, °C
t	Variable	Time, s
$\bar{u}$	Vector with δ and θ	Vector of the degrees of freedom in the compatibility equation
$U_{x}$	0 mm	Displacement in *x* direction
v	12 and 16 mm/s	Welding speed, mm/s
x	-	*x*-coordinate, transverse direction, mm
X	-	coordinate in the local heat source reference system, mm
y	-	*y*-coordinate, welding direction, mm
Y	-	coordinate in the local heat source reference system, mm
z	-	*z*-coordinate, mm
α	Equations (A9) and (A14)	Thermal diffusivity, mm^2^/s
$\alpha_{steel}$	Equation (A9)	Thermal diffusivity of steel, mm^2^/s
$\alpha_{Alu}$	Equation (A14)	Thermal diffusivity of aluminum, mm^2^/s
δ	Calculated	First degree of freedom of the compatibility equation, mm
Δt	1 s	Time step, s
$\Delta q_{p,i}^{x}$	Calculated	Plastic deformation in *x* direction at the current time step, mm
$\bar{\Delta q_{p}^{y}}$	Calculated	Vector of plastic deformation in *y* direction at the current time step, mm
λ	See below	Thermal conductivity, W/mm°C
$\lambda_{Alu}$	170 W/mm°C	Thermal conductivity of aluminum, W/mm°C
$\lambda_{Steel}$	44 W/mm°C	Thermal conductivity of steel, W/mm°C
ω	37 rad/s	Angular rotation speed, rad/s
$\sigma_{yield,Alu}^{}$	Equations (A10)–(A12)	Yield stress of aluminum, MPa
$\sigma_{yield,Steel}^{}$	Equations (A1)–(A4)	Yield stress of steel, MPa
$\sigma_{VM}$	Calculated	Von Mises stress, MPa
τ	20.8 and 15.6 s	Lag factor in Goldak’s equation, s
$\tau_{m}$	Calculated	Shear stress at tool-matrix interface, MPa
θ	Calculated	Second degree of freedom of the compatibility equation, rad
υ	$\upsilon_{steel}=0.28$ $\upsilon_{Alu}=0.34$	Poisson ratio

## Appendix A

(A1)
$$\sigma_{yield,Steel}=350\;for\;T\leq 420$$

(A2)
$$\sigma_{yield,Steel}=1.127E-5T^{3}-0.01942T^{2}+10T-1254.3\;for\;420<T\leq 700$$

(A3)
$$\sigma_{yield,Steel}=165.6-0.1037T\;for\;700<T\leq 1500$$

(A4)
$$\sigma_{yield,Steel}=0.01\;for\;1500<T$$

(A5)
$$E_{Steel}=206000-44.44T\;for\;T\leq 200$$

(A6)
$$E_{Steel}=26300-325T\;for\;200<T\leq 700$$

(A7)
$$E_{Steel}=1.229E-5-159.9T+0.05094T^{2}\;for\;700<T\leq 1200$$

(A8)
$$E_{Steel}=5000\;for\;1200<T$$

(A9)
$$\alpha_{Steel}=1.29E-5+2.218E-9T\;$$

(A10)
$$\sigma_{yield,Alu}=120-0.03392T\;for\;T\leq 300$$

(A11)
$$\sigma_{yield,Alu}=283-0.58T\;for\;300<T\leq 460$$

(A12)
$$\sigma_{yield,Alu}=69-0.107T\;for\;460<T\leq 638$$

(A13)
$$E_{Alu}=-0.1155T^{2}-41.06T+71000$$

(A14)
$$\alpha_{Alu}=2.188e-8T+2.25e-5$$
